# Myth or reality: the oxygen effect in rectal cancer radiotherapy

**DOI:** 10.3389/fonc.2025.1662791

**Published:** 2026-01-26

**Authors:** Zineb Dahbi

**Affiliations:** Radiation Oncology Department, Cheikh Khalifa University Hospital, Casablanca, Morocco

**Keywords:** oxygen effect, radiotherapy, radio-sensitivity, rectal cancer, survival

## Abstract

**Background:**

The oxygen microenvironment plays a crucial role in tumor radio-sensitivity, influencing the effectiveness of radiotherapy. The oxygen effect, which increases radiosensitivity in the presence of oxygen, has been extensively studied but not fully understood. This study investigates the relationship between hemoglobin variation, a surrogate marker for tissue oxygenation, and survival outcomes, in patients with locally advanced rectal cancer treated with neoadjuvant radiotherapy.

**Methods:**

In this retrospective study, we analyzed 97 patients with rectal adenocarcinoma treated with neoadjuvant pelvic radiotherapy between January 2016 and January 2024. Hemoglobin levels were measured before, during, and after radiotherapy, and hemoglobin variation (ΔHb) as a difference between maximum and minimum hemoglobin levels” was calculated. Active bone marrow (ABM) and low-density bone marrow (LDBM) were delineated for dosimetric analysis. Progression-free survival (PFS) was evaluated using Kaplan-Meier survival analysis, and the correlation between ΔHb, dosimetric data, and PFS was assessed using hazard ratios (HR) with a significance threshold of p < 0.05.

**Findings:**

The median volumes of LDBM and ABM were 114.6 cc and 765.4 cc, respectively. The maximal doses received by LDBM and ABM were 54.2 Gy and 52.2 Gy. The incidence of grade 3 and 4 anemia was 8.21%, with a mean delta hemoglobin (ΔHb) of 0.75%. No correlation was observed with grade 1–2 anemia at any dose level. A significant correlation was found between grade 3–4 anemia, ΔHb, and V40 doses for both LDBM and ABM (p-value < 0.05). Patients with ΔHb ≤ 1.5 demonstrated significantly better progression-free survival (PFS) compared to those with ΔHb > 1.5 (HR = 0.65, p = 0.0013). Dosimetric analysis revealed that higher doses to both ABM and LDBM regions did not correlate with improved PFS outcomes, while hemoglobin variation was a critical factor influencing survival. These results emphasize the role of hemoglobin variation and oxygenation in enhancing radio-sensitivity, particularly in active bone marrow.

**Interpretation:**

This study provides evidence that hemoglobin variation significantly influences survival outcomes in rectal cancer radiotherapy, supporting the clinical relevance of the oxygen effect. Hemoglobin monitoring and oxygenation strategies could be integrated into clinical practice to improve treatment efficacy, especially when considering dosimetric impacts on contoured bone marrow regions (ABM and LDBM). Future prospective studies are required to confirm these findings and explore interventions aimed at optimizing tissue oxygenation during radiotherapy.

## 1 Introduction

The oxygen microenvironment has a strong influence on how tumors evolve and respond to treatment. This phenomenon defines the oxygen effect, which refers to the increased radiosensitivity of free-living cells and organisms in the presence of oxygen compared to anoxic or hypoxic conditions, and it is explained by the oxygen fixation hypothesis, which proposes that radical-induced DNA damage can be permanently “fixed” by molecular oxygen, making the DNA damage irreparable (1). There are direct and indirect ways to calculate human tissue oxygenation, one of them being hemoglobin levels (2).

Understanding tissue oxygenation is crucial for optimizing radiotherapy. Hemoglobin levels, among other indicators, can provide insights into tissue oxygenation and, consequently, the potential impact of the oxygen effect on treatment outcomes. Despite extensive research, the clinical application of the oxygen effect remains debated (3, 4).

## 2 Aims

We aimed to determine the clinical relevance of the oxygen effect by correlating hemoglobin variation with tumoral response and survival in patients undergoing neoadjuvant radiotherapy for rectal cancer. We therefore tried finding a correlation between the hemoglobin levels during radiotherapy, tumoral response and overall survival in patients treated with pelvic radiotherapy for locally advanced rectal Cancer.

## 3 Methods

### 3.1 Study design and population

For this study, we reviewed patients with histologically confirmed rectal adenocarcinoma, treated in a single institution with neoadjuvant pelvic radiotherapy between January 2016 and January 2024. Total neoadjuvant treatment protocols were included. We excluded patients with metastatic disease, previous pelvic radiotherapy, concurrent malignancies, or hematological issues. No patient required granulocyte–monocyte colony stimulating factor, or received red blood cell or platelet transfusion during pelvic radiation. Hemoglobin variation was scored according to the Common Terminology Criteria for Adverse Events (CTCAE), version 7.0.

### 3.2 Study protocol and data collection

For each selected patient, we collected demographic data, tumor histological type, location of the tumor, circumferential clearance margins, TNM classification, in addition to treatments protocols (chemotherapy and radiotherapy) and survival outcomes. After surgery, the pathologic reports were analyzed to assess the pathologic response using the TRG scores by Dworak ranging from Grade 0 (no regression) to Grade 4 (complete regression).

- For dosimetric data of the radiotherapy treatment plan: based on radio anatomy guides, we proceeded to delineate 2 sets of Bone marrow volumes: 2

#### 3.2.1 Low-density bone marrow

Generally, appears more hypodense (less dense) on simulation CT images, as it contains a greater proportion of fat.

#### 3.2.2 Active bone marrow

**Figure 1:**
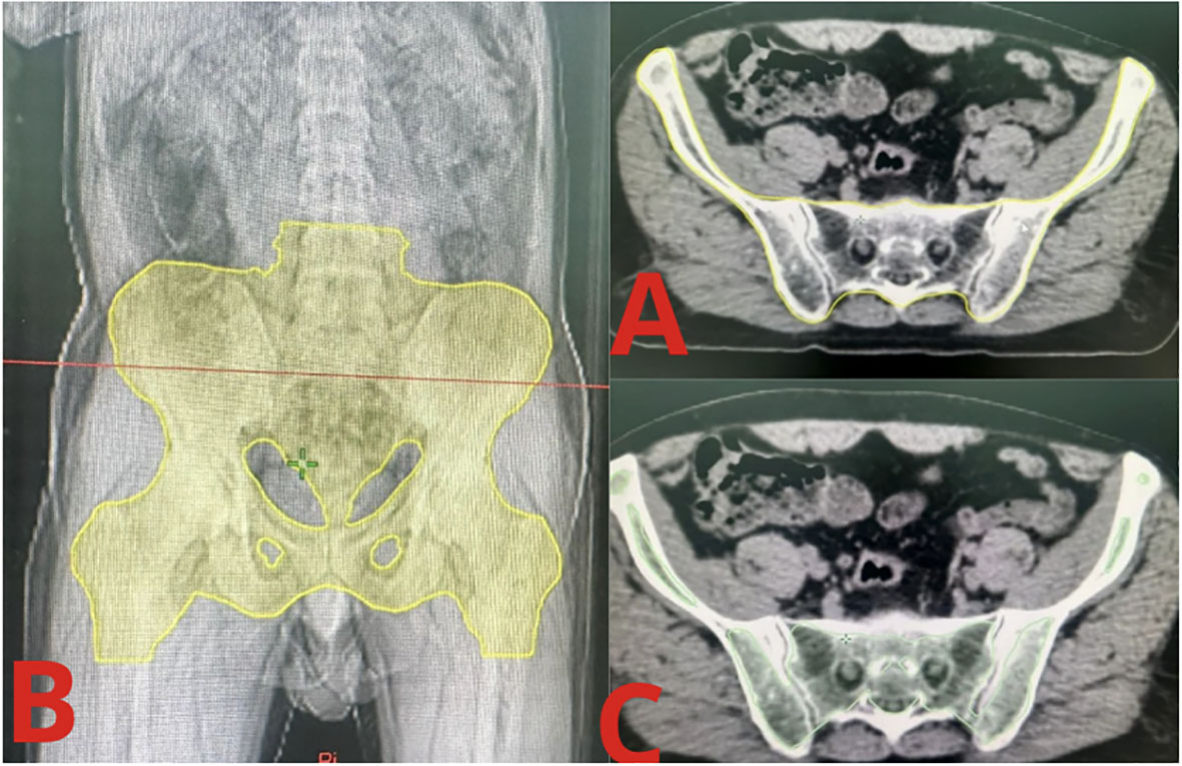
Contours of active and low-density bone marrow. **(A)** Yellow contours: Low-density Bone Marrow limits on axial simulation CT. **(B)** Yellow contours: Low-density Bone Marrow limits on reconstructed 2D image. **(C)** Green contours: Active Bone Marrow contours on axial simulation CT.

Appears more hyperdense (denser) as it contains a greater proportion of hematopoietic cells and less fat (**Figure 1**).

Without modification to the initial treatment plan for pelvic radiotherapy, the prescribed doses, mean and maximal doses to medullar cavity, along with volumes of both LDBM and ABM receiving 10,30 and 40Gy were obtained from our treatment planning system.

#### 3.2.3 For biological data

Hemoglobin levels were collected before, during pelvic radiotherapy and 3 and six months after the latter.

Using the CTCAE scale, anemia is classified as mild (grade 1) with hemoglobin levels between 10.0 and 11.9 g/dL, moderate (grade 2) with levels between 8.0 and 9.9 g/dL, severe (grade 3) with levels between 6.5 and 7.9 g/dL, and life-threatening (grade 4) with levels less than 6.5 g/dL.

The average maximum variation in hemoglobin levels (DeltaHb= Highest level minus lowest level) was defined as the largest change observed overtime.

### 3.3 Statistical analysis

Statistical analysis was performed with the Statistical Package for Social Sciences (SPSS, Chicago, IL) software package, version 17.0, Paired-sample comparisons were performed, and the paired t test was used. Regarding missing data, we employed multiple imputation to handle any missing values, ensuring that the results remained unbiased. A two-tailed P-Value less than 0.05 was considered as statistical significance in this study.

### 3.4 Ethical approval

Local Institutional ethics approval was granted to conduct this study.

## 4 Results

### 4.1 Patient characteristics

The study included 97 patients with a median age of 69 years. Fifty-four patients were male (54%), and the majority of patients (76%) had advanced clinical stages (stage III or above) at the time of diagnosis. General performance status was between 0 and 1 for 96.6% of patients. Tumor localization was in the lower rectum for 53% of patients and in the middle rectum for 47%. Tumor stages were classified as T3 (32.9%) and T4 (26.8%), with nodal involvement in 80.4% of cases. **Table 1** provides an overview of the patients’ clinical characteristics.

**Table 1:** Patient’s demographics and baseline characteristics.

Patients characteristics :		n	%
Median age (years)		69	
Sexe	Male	54	54,28%
	Female		
		43	45,72%
Distance from anal margin	<5 cm	52	53,44%
	5-10 cm	48	46,56%
Distance to MRF	<1mm	61	62,8%
Clinical T Stage	T1	13	13,4%
	T2	26	26,8%
	T3	32	32,9%
	T4	26	26,8%
Clinical N Stage	N0	19	19,55%
	N+	78	80,4%

The treatments administered to the patients included external radiotherapy, with a mean total dose of 50.2 Gy, ranging from 25 Gy to 66 Gy, and a mean dose per fraction of 1.9 Gy, varying from 1.8 Gy to 5 Gy. The radiotherapy techniques used were predominantly 3D (92.8%), while IMRT was used in 7.2% of cases. Brachytherapy was not used in any patient (0%). All patients (100%) received chemotherapy. Regarding specific neoadjuvant radiotherapy protocols, 35% (34 patients) were treated with the PRODIGE 23 protocol, 15% (15 patients) followed the RAPIDO protocol, and 49% (48 patients) received other neoadjuvant radiotherapy treatments. The mean duration of radiotherapy treatment was 39 days, and the median follow-up period was 72 months.

### 4.2 Correlation between dosimetric data for low-density bone marrow and active bone marrow

The median volumes of LDBM and ABM were 114.6 cc and 765.4 cc, respectively. The maximal doses received by LDBM and ABM were 54.2 Gy and 52.2 Gy, respectively. The mean V40 values were 410.8 cc for LDBM and 36.4 cc for ABM. Dosimetric data are summarized in **Table 2**.

**Table 2:** Correlation between dosimetric data of our study: Volume (in cc), maximal dose (Dmax), mean dose (Dmoy), V10 (volume in cc receiving 10Gy during the whole treatment), V30 (volume in cc receiving 30Gy during the whole treatment), V40 (volume in cc receiving 40Gy during the whole treatment).

	LDBM	ABM	p
Volume (cm3)	1144,6	765,4	0,034
Dmax (Gy)	54,2	52,2	0,12
D moy(Gy)	29,3	30,8	0,64
V10Gy	881,2	664,7	0,0145
V30Gy	68,7	486,1	0,02
V40Gy	410,8	36,44	0,001

### 4.3 Hemoglobin variation and histologic response

The incidence of grade 3 and 4 anemia was 8.21%, with a mean delta hemoglobin (ΔHb) of 0.75%. Hemoglobin levels and anemia grades during treatment were graded using the CTCAE criteria (grades I to IV), and histologic response was evaluated using the TRG score (1 to 4). No grade V toxicity was reported in our study. **Table 3** summarizes these findings.

**Table 3:** Combined hemoglobin levels and anemia grades during treatment graded following the CTCAE criterias (from grade I to IV) and histologic response graded following the TRG score (from 1 to 4).

		Number (n)	Percentage(%)
Hemoglobin levels	Anemia G1-G2	163	56,87
	Anemia G3-G4	24	8,21
	Median delta HB	0,75	
Histologic response	TRG score >3	79	81,4
	TRG score <3-4	18	18,5

### 4.4 Correlation between progression-free survival, hemoglobin levels, histologic response, and dosimetric data

A significant correlation was found between LDBM and ABM volumes and V10, V30, and V40 dose levels. However, no correlation was observed with grade 1–2 anemia at any dose level. A significant correlation was found between grade 3–4 anemia, ΔHb, and V40 doses for LDBM and ABM (p-value < 0.05).

**Figure 2:**
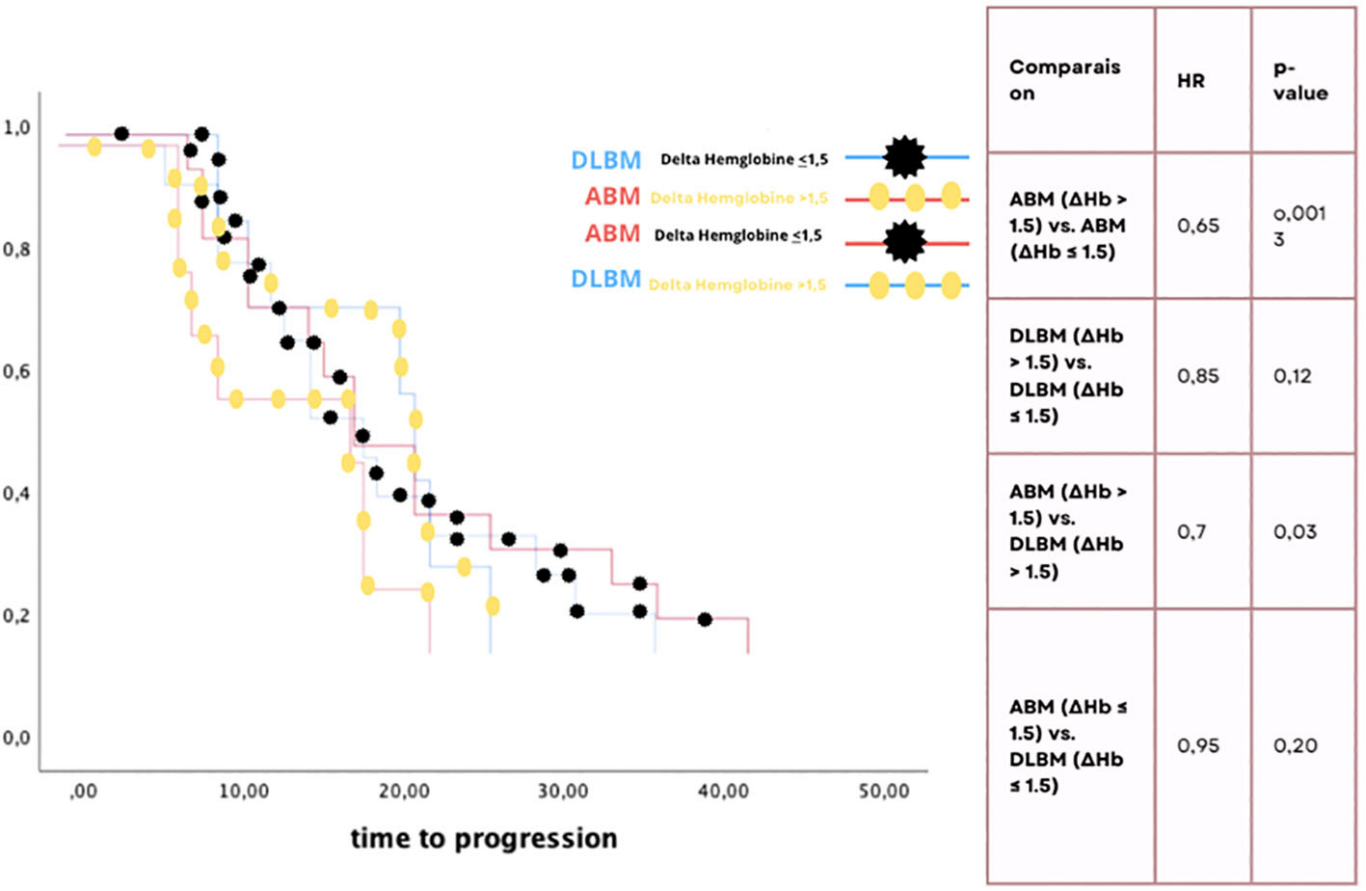
Kaplan-Meier survival curve and comparison of hemoglobin variation and bone marrow dosimetric impact on progression-free survival: ΔHb ≤ 1.5 show significantly improved PFS.

The Kaplan-Meier analysis showed that patients with active bone marrow (ABM) and a hemoglobin variation (ΔHb) less than or equal to 1.5 had significantly better progression-free survival (HR = 0.65, p = 0.0013). Additionally, patients with ΔHb ≤ 1.5, regardless of whether ABM or low-density bone marrow (DLBM) was used for contouring, had a significant survival advantage (HR = 0.7, p = 0.03). No significant differences were observed for patients with ΔHb > 1.5, regardless of bone marrow contouring method (**Figure 2**).

The multivariate analysis revealed that ΔHb remained a significant predictor of PFS after adjusting for other factors (hazard ratio [HR] = 0.68, 95% confidence interval [CI]: 0.50–0.92, p = 0.012). In contrast, age and tumor stage were not found to be statistically significant predictors of PFS in this cohort. Specifically, while higher ΔHb values (>1.5) were associated with poorer survival, this effect was adjusted for the influence of other covariates in the model.

These results highlight the impact of hemoglobin variation and bone marrow contouring method on survival outcomes, emphasizing the role of hemoglobin variation in predicting patient survival (**Table 4**).

**Table 4:** Correlation between dosimetric data, hemoglobin variation, and progression-free survival.

		P
Hemoglobil levels	Anemia G1-G2	0,15
	Anemia G3-G4	0,28
	Delta HB	0,003
Histologic response	TRG score>3	0,0028
	TRG score <3-4	0,325
Low denity BM	Dmax	0,853
	Dmoy	0,983
	V49Gy	0,622
Active BM	Dmax	0,948
	Dmoy	0,904
	V40Gy	0,997

## 5 Discussion

The oxygen effect has been a pivotal concept in radiobiology, influencing our understanding of tumor radiosensitivity in the context of hypoxia. Our study sought to explore the clinical applicability of this concept by assessing hemoglobin levels as a surrogate for tissue oxygenation and their correlation with oncological outcomes in patients undergoing neoadjuvant pelvic radiotherapy for locally advanced rectal cancer. The findings of this study provide insight into the potential role of the oxygen effect in optimizing radiotherapy, particularly through the lens of hemoglobin variation and its impact on bone marrow dosimetry.

We observed a significant correlation between hemoglobin variation (ΔHb) and progression-free survival (PFS), particularly in patients with active bone marrow (ABM). ΔHb affects PFS outcomes, highlighting the critical role of oxygenation in enhancing radiosensitivity. This aligns with the oxygen fixation hypothesis, wherein molecular oxygen aids in the fixation of radiation-induced DNA damage, rendering it irreparable and thereby enhancing the therapeutic efficacy of radiotherapy (1). Increased sensitivity to hemoglobin variation was observed in the contoured ABM region, which may reflect differences in radiosensitivity based on the delineation of bone marrow regions for dosimetric analysis. Conversely, low-density bone marrow (LDBM), which has a higher fat content, exhibited less of a survival benefit in response to hemoglobin variation. Interestingly, patients with lower hemoglobin variations (ΔHb ≤ 1.5) did not show significant differences in PFS, which raises questions about the threshold at which oxygenation impacts radiosensitivity. This observation could suggest that hemoglobin levels below a certain threshold fail to enhance oxygenation sufficiently to affect tumor radiosensitivity and treatment outcomes. Furthermore, the differential impact observed between ABM and LDBM highlights the heterogeneity of bone marrow compartments in their response to radiation and oxygenation, indicating that ABM may be a more reliable target for assessing the oxygen effect in clinical practice.

Interestingly, patients with lower hemoglobin variations (ΔHb ≤ 1.5) had significant differences in PFS, which raises questions about the threshold at which oxygenation impacts radiosensitivity. This observation could suggest that hemoglobin levels below a certain threshold fail to enhance oxygenation sufficiently to affect tumor radiosensitivity and treatment outcomes. Furthermore, the differential impact observed between ABM and LDBM highlights the heterogeneity of bone marrow compartments in their response to radiation and oxygenation, indicating that ABM may be a more reliable target for assessing the oxygen effect in clinical practice.

The relationship between tumor oxygenation, hemoglobin levels, and radiotherapy outcomes has been the subject of extensive research, yielding varying results. Advanced imaging to assess hypoxia and personalize treatment has been highlighted as essential, though direct measurement of the oxygen effect in clinical practice remains limited (2). While blood transfusions can correct anemia, they do not always result in significant improvements in survival or tumor response, suggesting that simply increasing hemoglobin levels may not translate to better outcomes (3). Similarly, prior meta-analyses have shown inconsistent results on hemoglobin’s influence on survival, with hazard ratios for overall survival (OS) ranging widely, emphasizing the complexity of this issue across different studies (3).

Our study supports findings that anemia or hypoxia during treatment can lead to worse outcomes (4–6). Anemia has been associated with poorer disease-free survival (DFS) and OS, and hypoxia has been linked to lower pathological response rates (5). These results align with our findings, which showed a significant correlation between hemoglobin variation (ΔHb) and progression-free survival (PFS), especially in patients with active bone marrow (ABM) and ΔHb less than 1.5 (6–9). Additionally, our results are in line with research showing that higher pre-treatment hemoglobin levels are associated with better tumor downstaging and higher TRG scores (9–11). Together, this literature (**Table 5**) highlights the crucial role of oxygenation in improving radiotherapy outcomes, reinforcing our study’s conclusion that hemoglobin variation and bone marrow type are key factors in the efficacy of rectal cancer treatment.

**Table 5:** Summary of key findings from relevant literature and current study on the impact of hemoglobin levels and tumor hypoxia in cancer radiotherapy.

Study	Study type	Key fundings
Shih et al (2019)	Review	No direct quantifiable data
Hoff et al (2012)	Observational study	OS: NS),TRG: NS
Lee et al (2011)	Retrospective Study	HR OS: 1.76 (negative impact of anemia),HR DFS: 2.03
Varlotto et al (2005)	Metanalysis	OS HR ranged: 0.65-1.25 (inconsistent results)
Nordmark et al (2005)	Retrospective study	pCR lower in hypoxic tumors
Adam et al (1998)	Prospective study	Hb > 12 g/dL correlated with better tumor downstaging (TRG significantly higher)
Our study (dahbi et al)	retrospective	HR PFS: 0.65 (p < 0.05 for ABM patients with delta Hb > 1.5)

### 5.1 Study limitations

Our study’s retrospective design and relatively small sample size limit the generalizability of the findings. Hemoglobin levels were measured at set intervals, which may not fully capture real-time fluctuations in oxygenation. Future prospective studies with larger cohorts are needed to validate these results. Additionally, exploring the impact of oxygen-enhancing interventions, such as blood transfusions or erythropoiesis-stimulating agents, could offer further insights into improving radiotherapy outcomes.

### 5.2 Research Implications

Future research should focus on real-time hemoglobin monitoring during radiotherapy to better understand oxygenation’s role in tumor response. There is also potential to explore oxygen-enhancing strategies like hyperbaric oxygen or pre-treatment interventions (e.g., transfusions) to improve outcomes. Studies assessing other oxygenation biomarkers through advanced imaging could further personalize radiotherapy protocols for rectal cancer patients.

## 6 Conclusion

Our findings suggest that hemoglobin variation significantly impacts survival outcomes in rectal cancer patients undergoing radiotherapy. By monitoring hemoglobin levels during treatment and considering oxygen-enhancing interventions, such as blood transfusions or oxygen therapy, it may be possible to improve treatment outcomes. These results emphasize the importance of personalized radiotherapy approaches that take into account bone marrow dosimetry (both ABM and LDBM) to optimize tumor oxygenation and enhance radiosensitivity. This opens new avenues for research into integrating oxygenation-enhancing strategies with tailored radiotherapy protocols to improve the therapeutic ratio in rectal cancer.

## Data Availability

The raw data supporting the conclusions of this article will be made available by the authors, without undue reservation.
